# Arsenite oxidase in complex with antimonite and arsenite oxyanions: Insights into the catalytic mechanism

**DOI:** 10.1016/j.jbc.2023.105036

**Published:** 2023-07-11

**Authors:** Filipa Engrola, Márcia A.S. Correia, Cameron Watson, Carlos C. Romão, Luis F. Veiros, Maria João Romão, Teresa Santos-Silva, Joanne M. Santini

**Affiliations:** 1 UCIBIO – Applied Molecular Biosciences Unit, Department of Chemistry, School of Science and Technology, NOVA University Lisbon, Caparica, Portugal; 2 Associate Laboratory i4HB - Institute for Health and Bioeconomy, School of Science and Technology, NOVA University Lisbon, Caparica, Portugal; 3 Division of Biosciences, Institute of Structural and Molecular Biology, University College London, London, United Kingdom; 4 ITQB NOVA, NOVA University Lisbon, Oeiras, Portugal; 5 Centro de Química Estrutural, Institute of Molecular Sciences, Lisboa, Portugal; 6 Departamento de Engenharia Química, Instituto Superior Técnico, Universidade de Lisboa, Lisboa, Portugal

**Keywords:** arsenite oxidase, enzyme mechanism, DFT calculations, X-ray crystallography, molybdenum enzyme, arsenic, antimony

## Abstract

Arsenic contamination of groundwater is among one of the biggest health threats affecting millions of people in the world. There is an urgent need for efficient arsenic biosensors where the use of arsenic metabolizing enzymes can be explored. In this work, we have solved four crystal structures of arsenite oxidase (Aio) in complex with arsenic and antimony oxyanions and the structures determined correspond to intermediate states of the enzymatic mechanism. These structural data were complemented with density-functional theory calculations providing a unique view of the molybdenum active site at different time points that, together with mutagenesis data, enabled to clarify the enzymatic mechanism and the molecular determinants for the oxidation of As(III) to the less toxic As(V) species.

Arsenic and antimony are two metalloids that, due to anthropogenic and natural causes (1, 2), pose an environmental threat and are considered by the World Health Organization as priority pollutants. Maximum drinking water recommended levels (10 ppb As, 20 ppb Sb) (3) are exceeded in many places throughout the planet with no remediation solution simultaneously effective, clean, and economically sustainable (4, 5, 6, 7). Chemically, both elements share numerous similarities: in aqueous solution, oxidation states III and V are the most common, speciating as oxyanions of arsenite/antimonite (As^III^(OH)_3_/Sb^III^(OH)_3_), thermodynamically favored in anoxic environments, and arsenate/antimonate ([As^V^(OH)_2_O_2_]^-^/[Sb^V^(OH)_2_O_2_]^-^/[Sb^V^(OH)_6_]^-^), favored under oxygenated conditions (5, 8, 9). Both elements have relatively high redox potentials, E^0^′ +140 mV and +94 mV for As^III^/As^V^ and Sb^III^/Sb^V^, respectively, which explains the existence of both oxidized and reduced forms in environmental and biological conditions (10, 11). The reduced forms of As and Sb are harder to remove from soils and water and are considered more toxic to living organisms than the oxidized ones (8, 10).

Arsenite oxidase (Aio) is an ancient bioenergetic enzyme present in microbes since the early stages of life on Earth, with its origin dated prior to the evolutionary split of Archaea and Bacteria (12). Widespread among prokaryotes, it has been identified in 78 different bacterial strains (13, 14) and purified from several microorganisms (15, 16, 17, 18, 19, 20). The Aio from *Pseudorhizobium banfieldiae* str. NT-26 (previously *Rhizobium* sp. str. NT-26) (NT-26 Aio) has been tested as a biosensor for arsenite (21, 22) but optimization is required.

Aio belongs to the dimethyl sulfoxide reductase family of molybdopterin (Moco)-dependent enzymes, with the Mo atom coordinated by two molybdopterin guanine dinucleotides (MGD) (23). The enzyme catalyzes the 2-electron oxidation of As^III^ as well as of Sb^III^, albeit with pronounced differences in reaction kinetics: Sb^III^ salts are oxidized around 6500 times slower than the corresponding As^III^ salt (20, 24).

To date, the only 3D structures available are those from NT-26 Aio (PDB 4AAY/5NQD) and from *Alcaligenes faecalis* Aio (*Af* Aio) (PDB 1G8K/1G8J) (25, 26, 27). The enzymes differ in terms of their quaternary structures (Fig. S1) but exhibit overall similar structures despite only 48% sequence identity; they superimpose with RMSD of 1.84 Å for Cα of 948 matching residues. The large subunit, AioA, contains the catalytic Moco and one [3Fe-4S] cluster, while the smaller subunit, AioB, harbors a Rieske [2Fe-2S] cluster (27). Aio is unique when compared to other members of the dimethyl sulfoxide reductase family: (i) the Mo ion is not coordinated to an amino acid residue side chain; (ii) according to crystallographic and extended X-ray absorption fine structure (EXAFS) data, Mo is bound to one/two oxo ligand(s) in addition to the two MGD dithiolenes; and (iii) it harbors a [3Fe-4S] and a Rieske cluster, instead of the typical [4Fe-4S] and/or [2Fe-2S] clusters (Fig. 1). The unusual features of this enzyme and its importance in bioremediation/biosensing aroused the interest of the scientific community in the last 2 decades (28, 29, 30, 31). A proposed mechanism of arsenite oxidation at the Mo center was suggested based on the X-ray structures of the free enzyme, cyclic voltammetry, EXAFS, and density-functional theory (DFT) calculations (25, 26, 31, 32). To further clarify how arsenite and antimonite are oxidized, we solved and analyzed the crystal structures of functionally relevant As–Sb complexes of NT-26 Aio and *Af* Aio. These data were integrated with site-directed mutagenesis and DFT calculations, allowing us to disclose the catalytic mechanism of Aio and the structural determinants, paving the way for future biotechnological applications.

**Figure 1:**
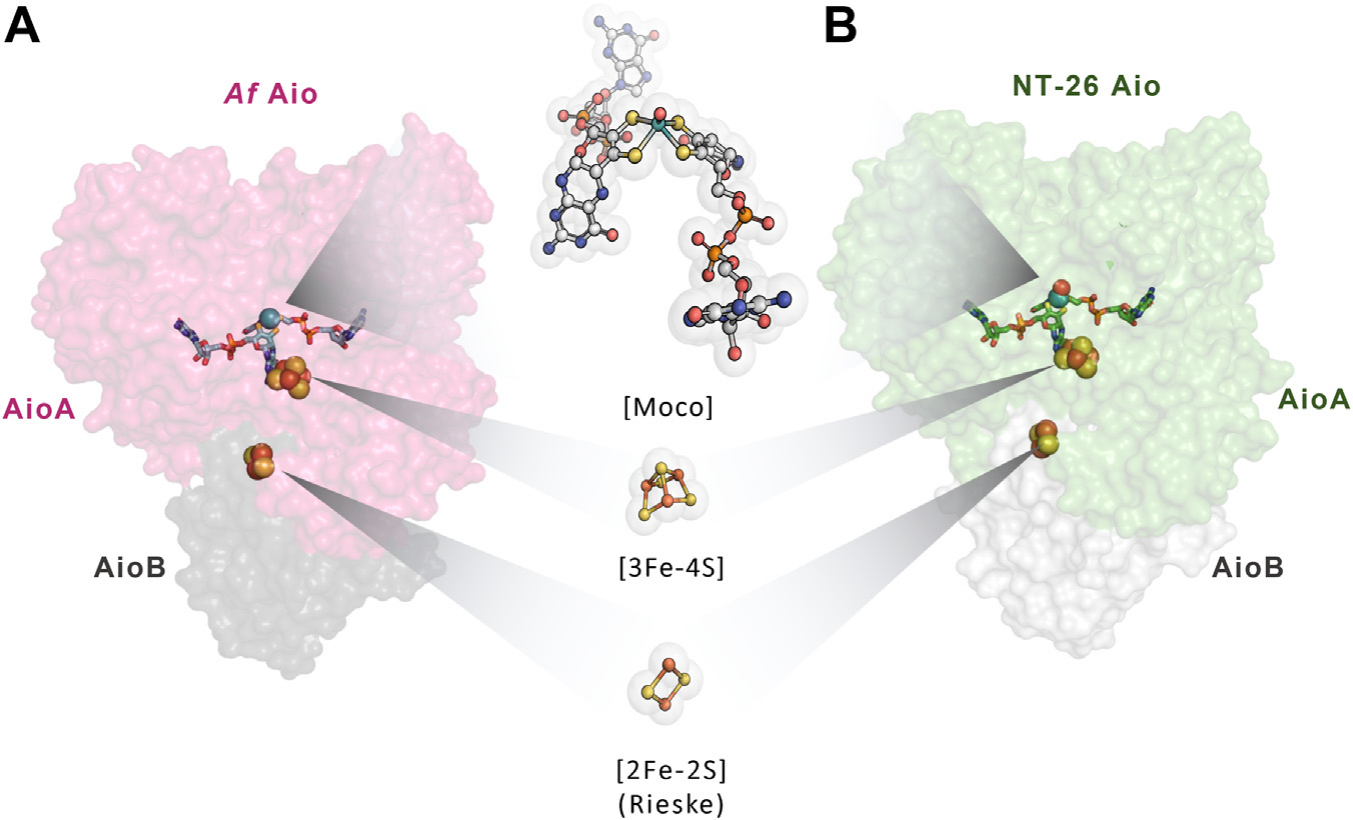
**Structure of Af Aio and NT-26 Aio enzymes highlighting the Moco and the iron-sulfur cluster cofactors.** AioA subunit harbors the Moco cofactor—with Mo bound to one/two oxo ligand(s), in addition to the two molybdopterin guanine dinucleotide (MGD) dithiolenes—and a [3Fe-4S] cluster. (*A*) Af AioA PDB ID 1G8K (26) in *pink* and (*B*) NT-26 AioA in *green* (PDB ID 5NQD [27]). AioB subunit harbors a Rieske iron-sulfur center (Af AioB in *grey* and NT-26 AioB in *white*).

## Results and discussion

### Overall structures of Aio–oxyanion complexes and the catalytic pocket

Four Aio–oxyanion complexes were obtained using different crystallization conditions and soaking protocols, varying the soaking time and the substrate—arsenite and antimonite salts—after enzyme activation with potassium ferricyanide (32) (crystallization details in Table S1). The high-resolution data (Table S2) revealed unusual coordination modes of As/Sb to the Moco center, corresponding to putative reaction intermediates. As in the published free structures (4AAY, 5NQD, and 1G8K (25, 26, 27)), the four NT-26 and *Af* Aio complexes crystallized as a dimer of heterodimers ([αβ]_2_) (Fig. S1, Table S3) with overall structures very similar to the ligand free forms. In all protein–ligand complexes, the Moco active site is at the bottom of a hydrophilic funnel-like cavity and the Mo atom adopts a five- or six-coordinated geometry: four sulfur atoms from the two MGD dithiolenes and one or two oxo-ligands, in a square pyramidal or a trigonal prismatic geometry, respectively (Fig. 1).

**Figure 2:**
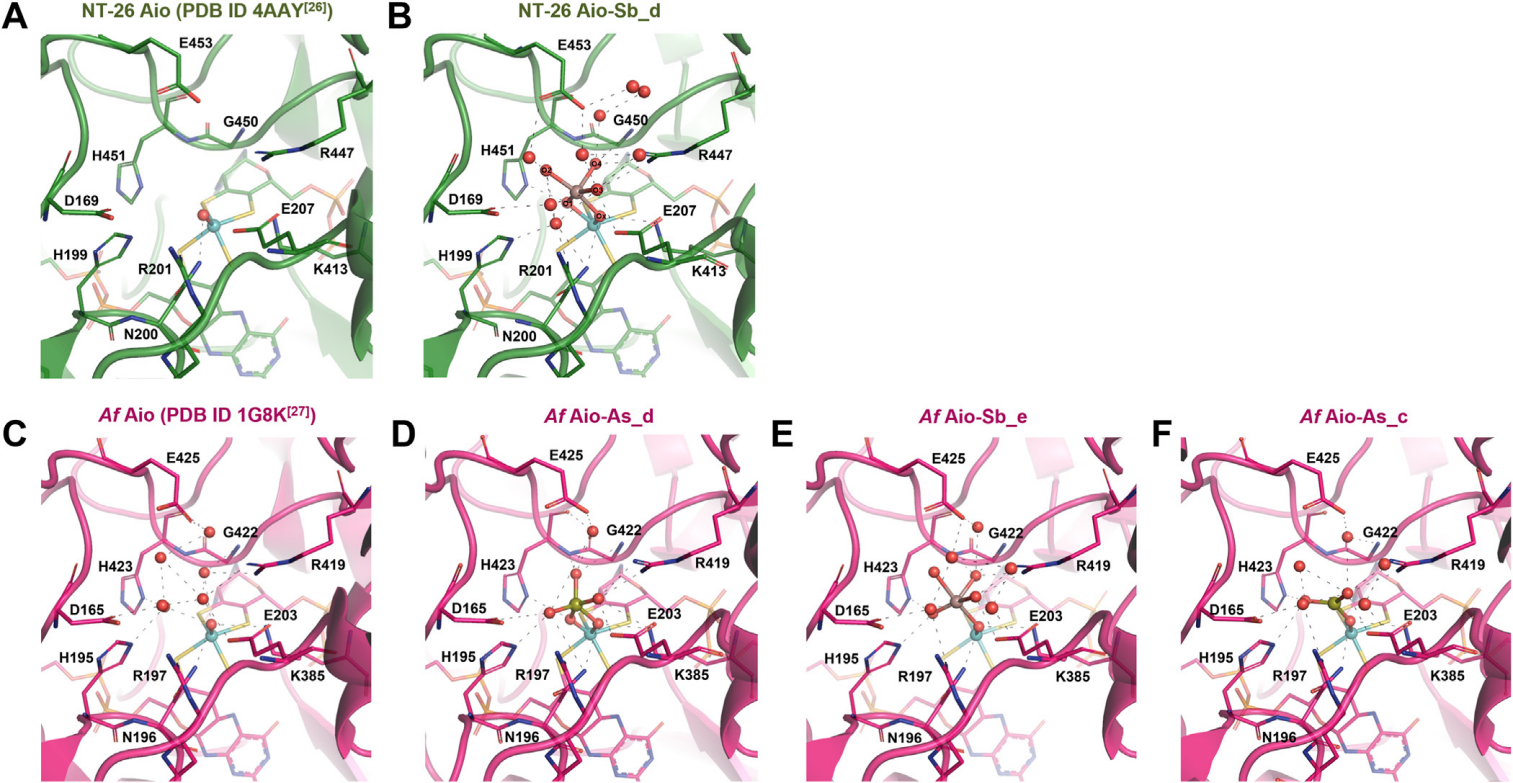
**Close-up view of the active site of the free and As/Sb oxyanion-bound forms of Aio.***A* and *C* correspond to ligand-free Aio, while *B* and *E* correspond to Sb-complex and D and F to As-complex: (*A*) NT-26 Aio (chain C) (PDB ID 4AAY), 2.7 Å resolution (25); (*B*) NT-26 Aio-Sb_d (chain E), 1.84 Å resolution; (*C*) Af Aio (chain A) (PDB ID 1G8K), 1.64 Å resolution (26); (*D*) Af Aio-As_d (chain E), 1.44 Å resolution; (*E*) Af Aio-Sb_e (chain A), 1.84 Å resolution; (*F*) Af Aio-As_c (chain E), 1.57 Å resolution. Images were prepared using PyMOL (34).

**Figure 3:**
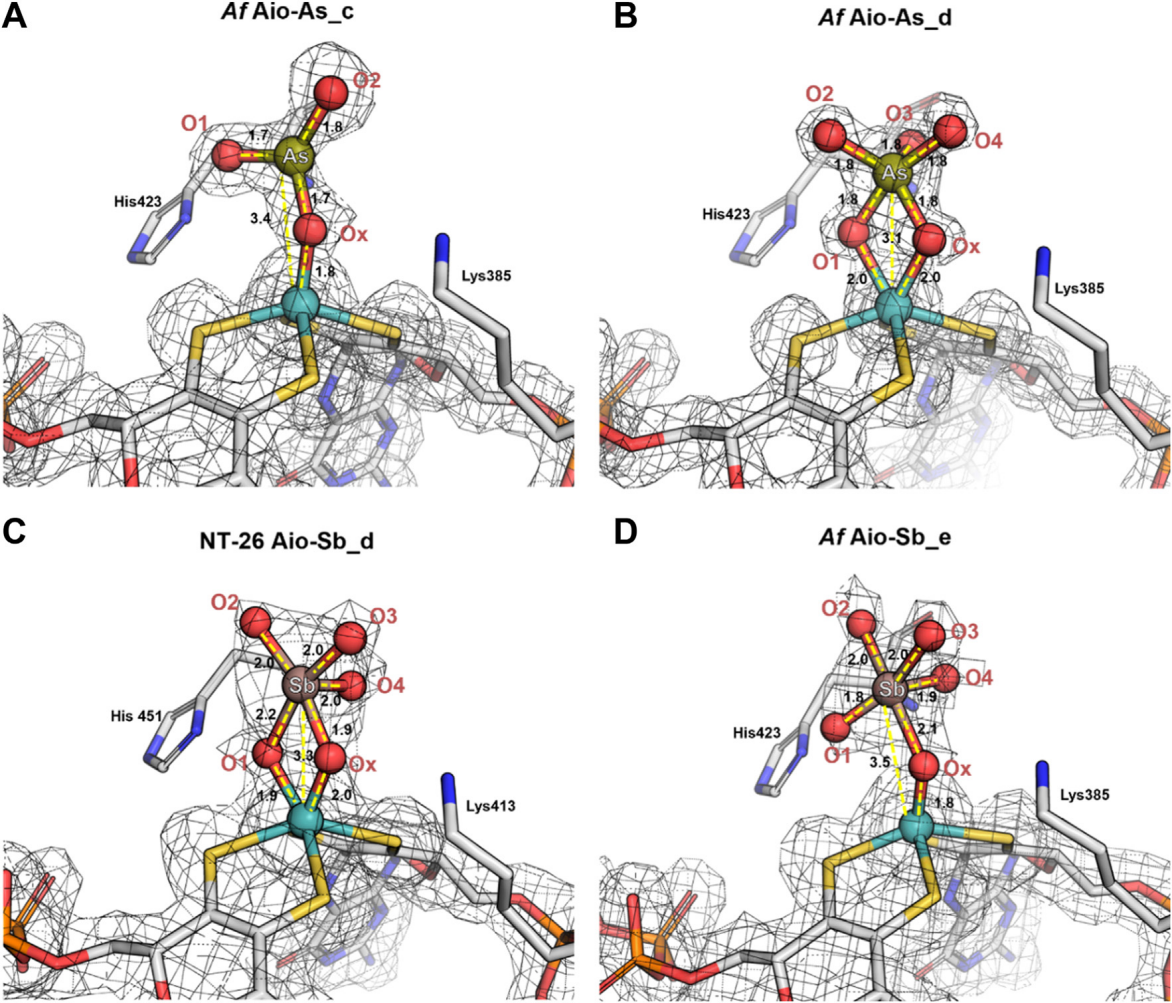
**Close up of the *Af* Aio and NT-26 Aio active sites in complex to As/Sb ligands.***A*, *Af* Aio-As_c (chain *E*), 1.57 Å resolution; (*B*) *Af* Aio-As_d (chain *E*), 1.44 Å resolution; (*C*) NT-26 Aio-Sb_d (chain *A*), 1.84 Å resolution; (*D*) *Af* Aio-Sb_e (chain *E*), 1.84 Å resolution. 2*F*_*o*_*-F*_*c*_ map contoured at 1σ. Distances are in Å. Images were prepared using PyMOL software (34).

In all structures, the presence of As or Sb at the enzyme active site is unambiguous, directly interacting with Mo *via* bridging oxygens (Figs. 2 and 3). At the second coordination sphere are several highly conserved residues: Asp169, His199, Arg201, Glu207, Lys413, Arg447, His451, Glu453 (NT-26 Aio numbering). Some of these residues (His199, Glu207, Arg447, His451) have been suggested as pivotal for catalysis (25, 26, 33) since they form direct hydrogen bonds with the Mo=O ligand (Fig. 2) while others interact with the substrate *via* a conserved network of water molecules. The two acidic residues Asp169 and Glu453 are located at the entrance of the highly solvated substrate funnel that leads to the Mo site, and their mutation to alanine resulted in a decrease of specific activity of 30 and 65%, respectively (Table S4; Fig. 2). Both residues are not H-bonded to residues of the catalytic site, only to water molecules that in turn make H-bonds with the Sb/As-oxyanions bound to Moco. These residues sit at *ca* 4.2 Å from O2 and O3 atoms and >5 Å from Sb (NT-26 Aio-Sb_d; Fig. 2*C* for reference). Since these residues are located at the entrance of the substrate tunnel, they are more exposed to solvent than those closer to the active site, as can be seen by the solvent-accessible surface area (SASA) (Table S5 (34)). Residue Asp169 has a SASA of >250 Å^2^ and Glu453 of >280 Å^2^, considerably larger than His451, at the first coordination sphere of Mo, with SASA of *ca* 140 Å^2^ (see Fig. 2*C* for reference). This indicates that, although these residues do not contact directly with the As/Sb ligands, they influence catalysis by stabilizing water molecules conserved in all structures determined that interact with the substrates through H-bonds (conserved in all structures determined). Interestingly, three water molecules found in the free *Af* Aio structure (1G8K) occupy the substrate-binding site as previously anticipated by Ellis (26) (Fig. 2).

Although As and Sb have been refined with partial occupancy, the corresponding B factors are in the same range as those of surrounding atoms (occupancy refinement was done iteratively using Refmac and Phenix software and by visual inspection 2*F*_*o*_*-F*_*c*_, *F*_*o*_-*F*_*c*_, and anomalous difference Fourier maps, and B factors; Table S6). The variation in occupancy of the As/Sb atoms in the structures can possibly be explained by differences in the crystallization and soaking conditions, and the dynamics of the reactions. It is known that the solubility and, thus, availability of As^III^ and Sb^III^ ions in sodium arsenite and antimonyl tartrate salts are pH-dependent, hence influenced by the different crystallization solutions used (35). Also, diffusion through the crystal channels is a limiting factor for reactivity and not all available catalytic sites are equally exposed in the crystal matrix. The four refined structures are now described individually. It should be stressed that, as expected, the four Aioα polypeptide chains (A, C, E, G) present slight variability in bond distances at the active sites (Tables S7 and S8) but a clear pattern is observed in the different cases.

### Structure of *Af* Aio-As_c

**Figure 4:**
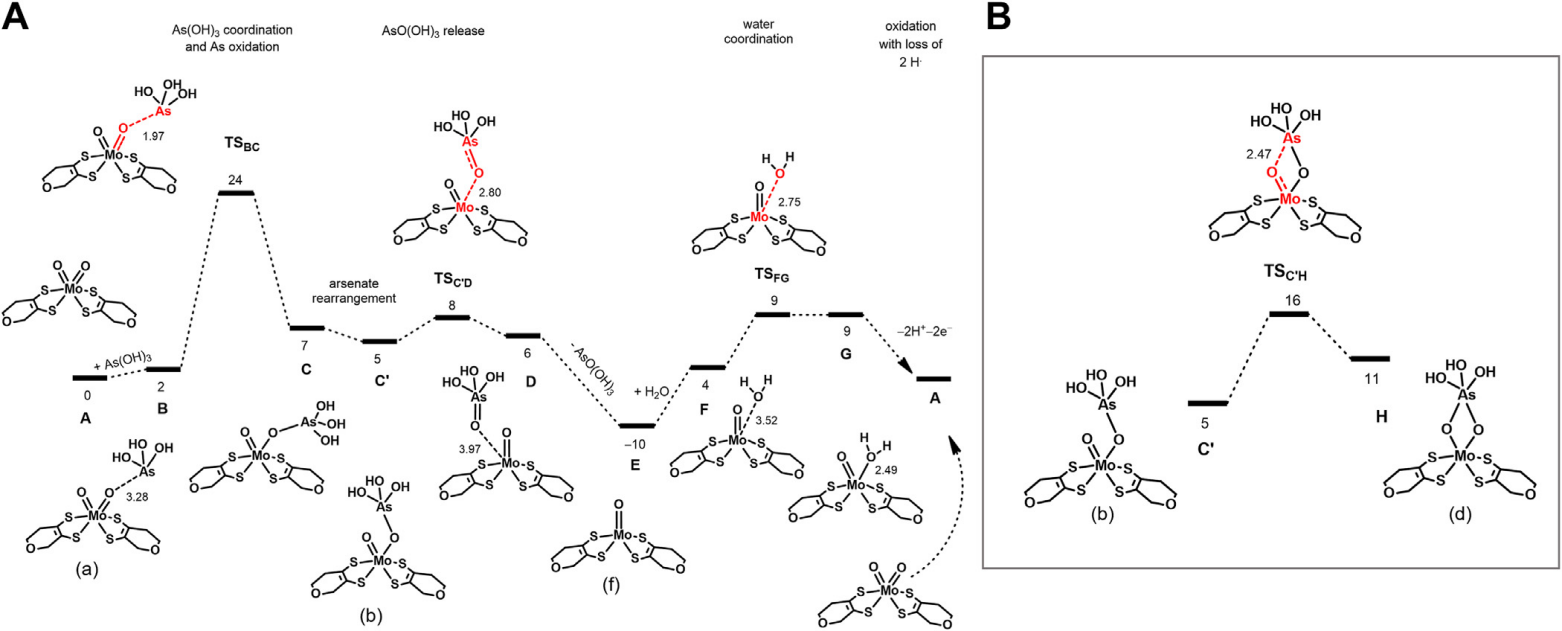
**DFT calculations of Aio mechanism**. *A*, DFT calculated fee energy profile (kcal/mol) for the As(OH)_3_ oxidation reaction. *B*, Conversion between μ1- and μ2-oxo bridged arsenate intermediates. Distances in Å. DFT, density-functional theory.

In this structure, the As ion is bound to Mo *via* one μ-oxo bridge (Mo-Ox-As) and coordinated to two other oxo/hydroxo groups (O1 and O2) (Figs. 3*A* and 4 and Fig. S3 showcasing anomalous map). The oxygen Ox shared by Mo and As is apical to Mo, at 1.7 Å from Mo, and 1.7 to 1.8 Å from As (distance range in the four molecules of the asymmetric unit (Table S7)). In this structure, the Mo is pentacoordinated and the As and Mo ions are 3.3 Å apart, in agreement with NT-26 Aio-As EXAFS data (32). In *Af* Aio-As_c, the As atom is not apical to Mo and the Mo-O-As bond is not linear but bent (about 140°) with the AsO_3_ group slightly over the MGD-P dithiolene (Fig. 3*A*).

### Structures of NT-26 Aio-Sb_d and *Af* Aio-As_d

In these structures, the Mo atom is *cis* coordinated to two oxygen atoms (O1 and Ox), shared with As/Sb (Fig. 3, *B* and *C*). The Mo-O single bond distances are ∼2 Å (varying 1.77–2.29 Å in the four molecules of the asymmetric unit (Table S7)). In both structures, the As/Sb atoms are sitting vertically to the Mo dithiolene plane, at *∼*3.1 Å for the As–Aio complex and ∼3.3 Å for the Sb–Aio complex (Fig. 3; Tables S7 and S8) and equidistant to the two pterins: the four dithiolene sulfur atoms are at 4.5 to 4.8 Å from As and 4.6 to 5.2 Å from Sb. The bond lengths within the As/Sb oxyanions (As/Sb to O1, Ox, O2, O3, and O4) are very similar in the four molecules (Table S7). The crystal structure of a polyoxometalate containing μ-oxo–bridged Sb and Mo (CSD YORPUS (36)) (Fig. S2) shows a similar geometry and bond distances as the intermediate structure (NT-26 Aio-Sb_d) now reported.

### Structure of *Af* Aio-Sb_e

In this structure, the Sb atom is sitting vertically to Mo and coordinated to the oxo ligand (Mo-Ox-Sb) and to four additional oxo/hydroxo groups (O1, O2, O3, and O4) (Fig. 3*D*). Sb-O distances are ∼2 Å and Sb is ∼3.5 Å from the Mo (Table S6), closer to the MGD-P dithiolene. In contrast to the Aio_d intermediates, where the two bridging oxygens (O1 and Ox) are equidistant to Mo, in Aio-Sb_e, the Mo-O1 distance is too long to be considered a covalent bond (range 2.6–3.2 Å, Table S7). In this case, as in structure *Af* Aio-As_c, Mo is pentacoordinated, adopting a trigonal prismatic geometry.

### DFT calculations and reaction mechanism

**Figure 5:**
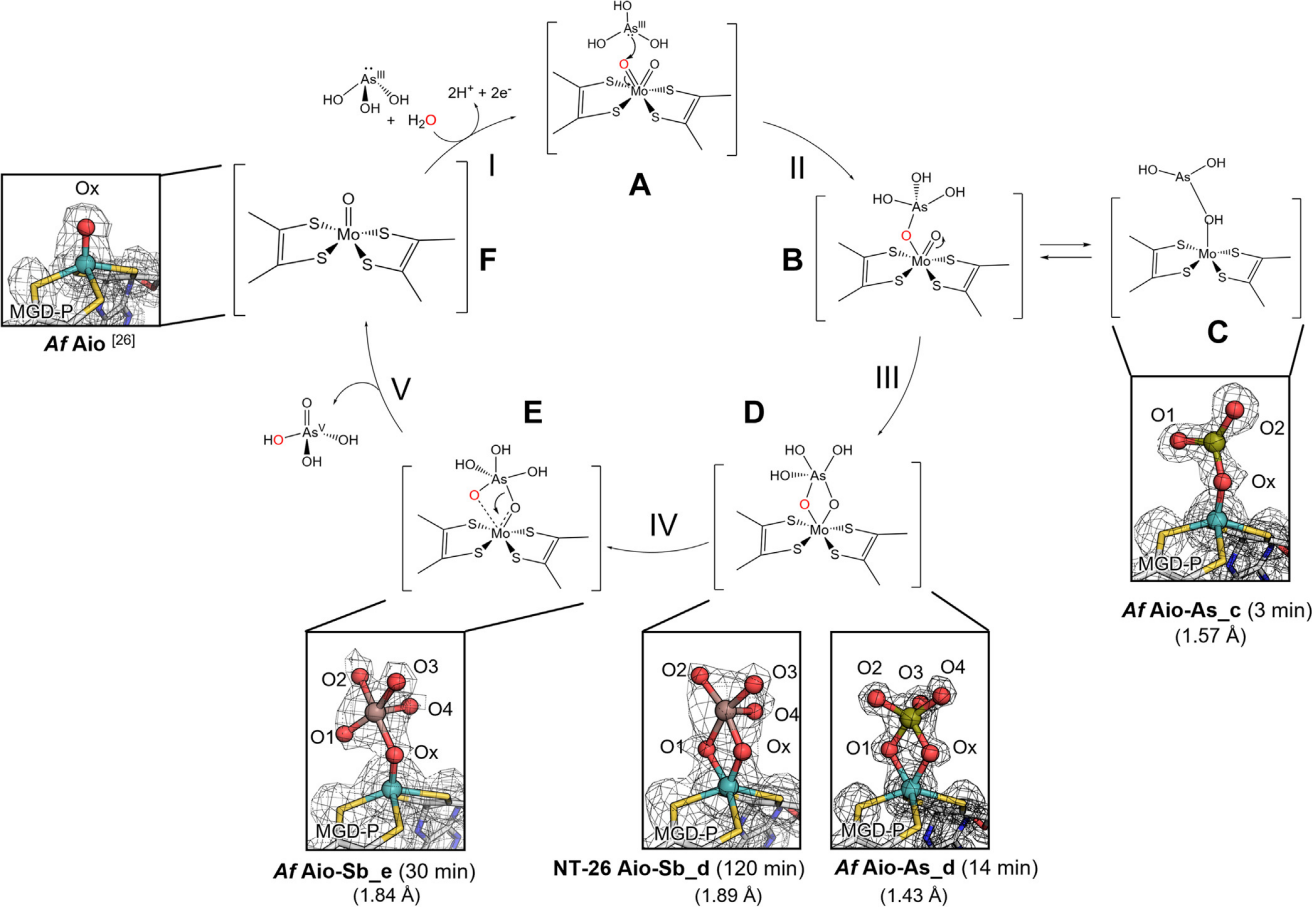
**New proposed reaction mechanism based on Aio-ligand crystal structures and DFT calculations.** The reaction initiation steps correspond to steps I and II, while the following steps (III and IV) are supported by the crystal structures here described (*C*, *D* and *E*); product release (step V) gives rise to Aio free structure (*F*), previously reported (1G8K [26]). Numbers in parenthesis correspond to the resolution of the diffraction data. DFT, density-functional theory.

So far, all ligand-free crystal structures of Aio show a single oxo group coordinated to the Mo atom (structure (F) in Fig. 5) probably resulting from X-ray photoreduction (to Mo^IV^), while the oxidized enzyme is proposed to have a di-oxo/hydroxo Mo coordination (Mo^VI^) (Fig. 5, structure (A)) (26). Considering the structures of the captured reaction intermediate forms here described, we suggest that the reaction cycle (Fig. 5) starts by a nucleophilic attack of the substrate arsenite to the oxidized 6-coordinated Mo (Mo^VI^), specifically *via* the nonspectator oxo ligand (O1, step I). This corresponds to the first step in the DFT calculated reaction profile (37), from A to C with a barrier of 24 kcal/mol (Fig. 4*A*).

The reaction proceeds with the As/Sb atoms moving apical to Mo from **C** to **C′** in the profile of Figure 4*A* (Fig. 5, step II). Interestingly, a structure with two symmetrical μ-oxo bridges (H) such as observed in the X-ray structures (D) (NT-26 Aio-Sb_d and *Af* Aio-As_d) is readily obtained from intermediate C**′** with a barrier of 11 kcal/mol (Fig. 4*B*, H). The μ-oxo bridges stabilize the substrate in the active site, enabling the oxo-transfer step, in which Mo-O1 bond is broken, giving rise to a single oxo coordinated Mo (Fig. 5 step V and structure (F), and E in Fig. 4*A* profile). This is a facile step with a calculated barrier of merely 3 kcal/mol (from C**′** to D, in Fig. 4*A*). The product displacement results in free arsenate/antimonate and a reduced Mo, with a single-oxo coordination, (Mo^IV^ = O/-OH). The addition of a water molecule and the concurrent transfer of two electrons and two protons to the iron-sulfur clusters regenerates the Mo^VI^ dioxo form and restarts the catalytic cycle (step I). This corresponds to the last part of the profile (Fig. 4*A*), from E back to A, and has a 19 kcal/mol barrier for water coordination. Considering the DFT calculations, the intermediate structure (C) (*Af* Aio-As_c) is not present along the mechanism and, thus, we have interpreted it as a side product and/or a dead-end structure.

## Conclusion

This work contributes to the clarification of the enzymatic reaction mechanism of the oxidation of As(III) to the less toxic As(V) revealing, for the first time, atomic resolution structures of putative intermediates bearing different As/Sb anions bound to the Mo active site. The Aio enzymatic mechanism relies on the formation of two symmetrical μ-oxo bridges, present in the oxidation of As(III) as well as of Sb(III). The oxyanions are held in the active site pocket by covalent bonding to the Mo cofactor as well as by an intricate network of H bonds with water molecules and charged side chains. In fact, site-directed mutagenesis revealed that two conserved acidic residues involved in this network are essential for catalysis even though they are located at the entrance of the catalytic funnel, at *ca* 8 Å from the Mo ion. The intermediate species here found are good evidence that reaction mechanisms often proposed based solely on theoretical calculations correspond to a simplification of the various states that arise during an enzymatic reaction, enlightening the importance of experimental structural data. This study provides high resolution data on a complex metalloprotein, combining crystallography, mutagenesis, and *in silico* calculations. The elucidation of the catalytic mechanism and the knowledge of the molecular determinants for catalysis are vital in the design of new and improved arsenite oxidases with higher stability and substrate specificity. These unique enzymes are very suitable candidates for biotechnological applications for the development of biosensors for As(III/V) and Sb(III/V) using engineered enzymes, as well as for bioremediation of these toxic species, where robust and reliable systems are still lacking (28, 30, 38, 39, 40).

## Experimental procedures

### Heterologous expression and purification of *Af* Aio and of NT-26 Aio—WT, D169A and E453A mutants

Expression of NT-26 and of *Af* Aio was done aerobically in *Escherichia coli* DH5α cells at 21 °C, for 24 h in LB media, containing 40 μM of IPTG; in the case of NT-26 Aio mutants, *E. coli* Tuner cells were used and 250 μM of IPTG was required for overexpression. The enzymes were purified using Ni^2+^ affinity, and in the case of NT-26 Aio WT, an additional step of size-exclusion chromatography was carried out to increase enzyme purity, as previously described (24, 26). The protein was concentrated in 50 mM Tris HCl pH 7.8, up to 20 mg mL^−1^, and stored at 4 °C.

### Crystallization and preparation of complexes of NT-26 Aio and *Af* Aio

WT enzymes were crystallized using the sitting drop vapor diffusion method. For NT-26 Aio, the crystallization drops were prepared using a ratio of 2 μl of protein and 1 μl of reservoir solution with 2 M ammonium sulfate as precipitant, 0.1 M Hepes pH 7.5, and 2% (v/v) PEG 400 (no mixing), as reported (27); in contrast, crystals of *Af* Aio were obtained using a 1 μl of protein and 1 μl of reservoir solution, using PEG-based conditions that differ from the published one (26) regarding pH and the presence of additives (Table S1; also, in this case, precipitant and protein solution were not mixed). For both NT-26 and *Af* Aio proteins, the best crystals appeared at 4 °C, as thin brownish plates, that grew to their maximum size (*ca* 0.2 × 0.2 × 0.05 mm) within approximately 2 weeks.

To obtain protein–ligand complexes, crystals were first soaked in a harvesting buffer containing a slightly higher concentration of the precipitating agent (ammonium sulfate for NT-26 Aio and PEG for *Af* Aio)—increment of 2% (v/v)—and 50 mM K_3_[Fe(CN)_6_] to oxidize the protein. Afterward, crystals were soaked in harvesting buffers containing 10 mM of one of the two substrates—potassium antimonyl tartrate trihydrate (Sb^III^) or sodium arsenite (As^III^)—using different soaking times (Table S1). The catalytic reaction was stopped upon flash-freezing the crystals in liquid nitrogen, having 20% (v/v) glycerol as cryoprotectant.

### X-ray data collection and structure determination

Diffraction data were collected at PXIII of the Swiss Light Source, at Biomax, MAX IV, and at XALOC, ALBA. The NT-26 Aio and the *Af* Aio crystals, soaked with As or Sb salts, diffracted to high resolution (ranging from 1.89 to 1.44 Å for the different complexes—see Table S2 for details). Data were processed using the XDS (https://xds.mr.mpg.de) and STARANISO (https://staraniso.globalphasing.org/cgi-bin/staraniso.cgi) program packages (41, 42). Data quality was analyzed using Aimless from the CCP4 package suite (43, 44), and structure determination was accomplished by molecular replacement using the ligand-free crystal structure of NT-26 Aio (PDB 5NQD (27)) and *Af* Aio (PDB 1G8K (26)) as search models. Interactive cycles of model building and refinement were performed with COOT (https://www2.mrc-lmb.cam.ac.uk/personal/pemsley/coot) (45), Refmac5 (https://www2.mrc-lmb.cam.ac.uk/groups/murshudov/content/refmac/refmac.html) (46), and PHENIX (https://phenix-online.org) software (47). To mitigate model bias and overfitting, the ligands were placed only on the latter stage of refinement, looking at the 2*F*_*o*_*-F*_*c*_, *F*_*o*_*-F*_*c*_, and anomalous difference Fourier maps. Data collection and refinement statistics are shown in Table S2.

### Activity assays of NT-26 Aio WT *versus* mutants D169A and E453

The NT-26 WT Aio and the D169A and E453A mutants were previously oxidized for 10 min with 10 mM potassium ferricyanide, and the excess of the oxidizing agent was removed using a PD-10 desalting column (GE Healthcare), according to the manufacturer's instructions, with 50 mM MES pH 5.5 as the elution buffer. The reactions were monitored at 600 nm following the reduction of the artificial electron acceptor 2,6-Dichlorophenolindophenol (considering Δε_red-ox(600nm)_ of 8.2 mM^−1^ cm^−1^) (48), using Na_3_As^III^O_3_ at 2.5 mM as the substrate. The results corresponding to three replicates for each protein (WT and mutants) are shown in Table S4.

### DFT calculations

All calculations were performed using the Gaussian 09 software package (http://gaussian.com/) (49). Geometry optimizations were obtained using the PBE0 functional without symmetry constraints and a basis set (b1) consisting of the Stuttgart/Dresden ECP basis set (50, 51, 52) to describe the electrons of Mo and As, with one *f*-polarization added for Mo (53) and one *d*-polarization function added for As (54); a standard 6-31G(d,p) basis set (55, 56, 57, 58, 59) was used for all other atoms. The PBE0 functional uses a hybrid generalized gradient approximation, including 25% mixture of Hartree-Fock (60) exchange with DFT (37) exchange-correlation, given by Perdew, Burke, and Ernzerhof functional (61, 62, 63). Transition state optimizations were performed with the Synchronous Transit-Guided Quasi-Newton method developed by Schlegel *et al.* (64, 65) following extensive searches of the Potential Energy Surface. Frequency calculations were performed to confirm the nature of the stationary points, yielding one imaginary frequency for the transition states and none for the minima. Each transition state was further confirmed by following its vibrational mode downhill on both sides and obtaining the minima presented on the energy profiles. The electronic energies (*E*_b1_) were converted to free energy at 298.15 K and 1 atm (*G*_b1_) by using zero-point energy and thermal energy corrections based on structural and vibration frequency data calculated at the same level.

Single point energy calculations were performed on the geometries obtained at the PBE0/b1 level using the M06 functional, the same basis for Mo and As, and a 6-311++G(d,p) basis set (66, 67, 68, 69, 70, 71, 72, 73, 74, 75) for the rest of the elements (basis b2). The M06 functional is a hybrid meta-generalized gradient approximation functional developed by Truhlar and Zhao (76), and it was shown to perform very well for the kinetics of transition metal molecules, providing a good description of weak and long-range interactions (77, 78). The free energy values presented (*G*_b2_) were derived from the electronic energy values obtained at the M06/b2//PBE0/b1 level (*E*_b2_) according to the following expression: *G*_b2_ = *E*_b2_ + *G*_b1_ – *E*_b1_.

Solvent effects (water) were considered in all calculations (including geometry optimizations) using the Polarizable Continuum Model initially devised by Tomasi et al (79, 80, 81, 82) with radii and nonelectrostatic terms of the SMD solvation model, developed by Truhler *et al.* (83).

The molybdopterin cofactor was replaced by a simplified dithiolate model in the calculations, for computational expediency. That model reproduces the pyran ring framework observed in the real molybdopterin cofactor (Fig. S4).

## Data availability

Data supporting this article are included within the main text and supporting information.

Atomic coordinates and structure factors for the crystal structures have been deposited at the Protein Data Bank (PDB), with the accession numbers: *Af* Aio-As_c: PDB ID 8CFF; NT-26 Aio-Sb_d: PDB ID 8CCQ; *Af* Aio-As_d: PDB ID 8CH9; *Af* Aio-Sb_e: PDB ID 8CGS.

## Supporting information

This article contains supporting information (25, 26, 27, 34, 36).

## Conflict of interest

The authors declare that they have no conflicts of interest with the contents of this article.

## Supporting information

Supporting Information

NT26Aio_Sb_d_PDB_ID_8CCQ_mtz.mtz

NT26Aio_Sb_d_PDB_ID_8CCQ.cif

AfAio_As_c_PDB_ID_8CFF.cif

AfAio_As_c_PDB_ID_8CFF_mtz.mtz

AfAio_Sb_e_8CGS_mtz.mtz

AfAio_Sb_e_PDB_ID_8CGS.cif

AfAio-As_d_PDB_ID_8CH9.cif

AfAio-As_d_PDB_ID_8CH9_mtz.mtz
