# Supplementary material for: Arsenite oxidase in complex with antimonite and arsenite oxyanions: Insights into the catalytic mechanism
Source: J Biol Chem. 2023 Jul 11;299(8):105036. doi: 10.1016/j.jbc.2023.105036 (PMC10448176; doi:10.1016/j.jbc.2023.105036)
Supplement: Supporting Information [file mmc1.docx]

**Supporting Information for “Arsenite oxidase in complex with antimonite and arsenite oxyanions - insights into the catalytic mecha nism”**

Filipa Engrola^a,b,#^, Márcia A. S. Correia^a,b,#^, Cameron Watson^c^, Carlos C. Romão^d^, Luis F. Veiros^e^, Maria João Romão^a,b,*^, Teresa Santos-Silva^a,b,*^, Joanne M. Santini^c,*^

## ﻿

^a^ UCIBIO – Applied Molecular Biosciences Unit, Department of Chemistry, School of Science and Technology, NOVA University Lisbon, 2819-516 Caparica, Portugal

^b^ Associate Laboratory i4HB - Institute for Health and Bioeconomy, School of Science and Technology, NOVA University Lisbon, 2819-516 Caparica, Portugal

^c^ Institute of Structural and Molecular Biology, Division of Biosciences, University College London, WC1E 6BT, United Kingdom

^d^ ITQB, NOVA University Lisbon, Av. da República, 2780-157 Oeiras, Portugal

^e^ Centro de Química Estrutural, Institute of Molecular Sciences, Departamento de Engenharia Química, Instituto Superior Técnico, Universidade de Lisboa, Av. Rovisco Pais, 1049 001 Lisboa, Portugal

* co-corresponding authors (mjr@fct.unl.pt; tsss@fct.unl.pt; j.santini@ucl.ac.uk)

^#^ both authors contributed equally to this work

**Table of Contents**

**Protein production, activity assays and X-ray Crystallography** 3

**Aio active site analysis** 7

**DFT supplementary information** 10

**Atomic coordinates of the optimized species** 10

**Figure S1.** NT-26 and *Af* Aio heterotetrameric arrangement 5

**Figure S2.** Sb-Mo polyoxometalate (CSD YORPUS) 9

**Figure S3.** *Af* Aio-As_c active site, chain E. Electron density map 2*Fo-Fc* and *mFo-DFc* anomalous map 10

**Figure S4.** Simplified molybdopterin cofactor model used in DFT calculations 11

**Table S1.** Crystallization and soaking conditions of Aio-As/Sb complexes 3

**Table S2.** Data collection and refinement statistics for the Aio complexes with Sb and As salts 4

**Table S3.** Analysis of protein-protein interactions of the reported Aio-free structures using PISA. 5

**Table S4.** Specific activity of NT-26 Aio WT and mutant enzymes 6

Table S5. Solvent-accessible surface area (Å^2^) of several conserved residues of NT-26 Aio-Sb_d structure……..6

**Table S6.** B factor analysis of all the atoms surrounding the Mo and As/Sb atoms and corresponding occupancy values 7

**Table S7.** Bond lengths around Mo and As/Sb atoms at the catalytic site of Aio structures 8

**Table S8.** Bond lengths between catalytic Mo and the coordinated sulfur atoms of the bis-MGD moieties of Aio-intermediate bound structures and in all molecules present in the asymmetric unit 8

**References** 19

# Protein production, activity assays and X-ray Crystallography

Table S1. Crystallization and soaking conditions of Aio-As/Sb complexes**.**

|  | **Crystallisation condition** | **Soaking with the substrate**  **(soaking time)** | |
| --- | --- | --- | --- |
| ***Af* Aio-As_c** | 10% v/v PEG 4000, 0.1 M sodium citrate pH 5.5 and 10% v/v isopropanol | sodium arsenite  (3 min) |  |
| ***Af* Aio-As_d** | 10% v/v PEG 6000, 0.01 M magnesium chloride | sodium arsenite  (14 min) |  |
| **NT-26 Aio-Sb_d** | 2 M ammonium sulphate, 0.1 M HEPES sodium salt pH 7.5 and 2% v/v PEG 400 | potassium antimonyl tartrate trihydrate  (120 min) |  |
| ***Af* Aio-Sb_e** | 15% v/v PEG 4000, 0.1 M sodium citrate pH 5.5 and 0.2 M ammonium acetate | potassium antimonyl tartrate trihydrate  (30 min) |  |

Table S2. Data collection and refinement statistics for the Aio complexes with Sb and As salts.

|  | NT- 26 Aio– Sb_d  (PDB ID 8CCQ) | | *Af* Aio–Sb_e (PDB ID 8CGS) | *Af* Aio–As_c  (PDB ID 8CFF) | *Af* Aio–As_d (PDB ID 8CH9) |
| --- | --- | --- | --- | --- | --- |
| Beamline | PXIII  (SLS) | | Biomax  (Max IV) | ALBA  (XALOC) | ALBA  (XALOC) |
| X-ray wavelength (Å) | | 0.978 | 0.918 | 0.979 | 0.979 |
| Resolution (Å) | | 49.30-1.89  (1.98-1.89) | 48.47-1.84 (1.87-1.84) | 107.43-1.57 (1.69-1.57) | 65.39-1.43 (1.56-1.43) |
| Space Group | | P22_1_2_1_ | P1 | P1 | P1 |
| Unit cell parameters | |  |  |  |  |
| a, b, c (Å) | | 141.5, 148.4,  232.7 | 90.4, 109.2, 117.3 | 90.3, 108.9, 116.9 | 90.3, 109.9, 117.4 |
| α, β, γ (^o^) | | 90.0 | 97.7, 90.0, 96.3 | 97.5, 90.2, 96.1 | 98.3, 89.9, 96.6 |
| No. of molecules per asymmetric unit | | 4 | 4 | 4 | 4 |
| No. of observed reflections | | 4 881 508  (230 091) | 737 465  (36 892) | 1 582 381  (75 555) | 2 053 159  (97 140) |
| No. of unique reflections | | 354 714  (17 429) | 365 490  (17 998) | 452 928  (22 648) | 594 307  (29 716) |
| Completeness [spherical] (%) | | 100.0 (100.0) | 95.3 (94.8) | 73.2 (18.4) | 73.0 (16.8) |
| Completeness [ellipsoidal] (%) | | - | - | 89.4 (52.3) | 88.2 (46.3) |
| <*I/*σ*(I)*> | | 13.1 (1.8) | 4.2 (1.7) | 5.6 (1.6) | 9.4 (1.7) |
| *R_pim_* (%)* | | 5.9 (16.5) | 6.1 (2.7) | 0.094 (0.312) | 0.101 (0.84) |
| *CC ½* | | 0.997  (0.511) | 0.993  (0.848) | 0.989  (0.780) | 0.997  (0.589) |
| Multiplicity | | 13.8 (13.2) | 2 (2) | 3.5 (3.3) | 3.5 (3.3) |
| Solvent content (%) | | 55.9 | 54.1 | 53.2 | 53.7 |
| Refinement | |  |  |  |  |
| *R_work_* (%)** | | 15.50 | 15.4 | 16.80 | 17.10 |
| *R_free_* (%)*** | | 20.03 | 18.9 | 20.40 | 0.20 |
| RMSD | |  |  |  |  |
| Bond lengths (Å) | | 0.018 | 0.036 | 0.009 | 0.015 |
| Bond angles (^o^) | | 2.188 | 2.060 | 1.624 | 1.9700 |
| Ramachandran plot (%)  Residues in favoured regions  Residues in allowed regions  Residues in disallowed regions | |  |  |  |  |
|  |  | 95.88 | 96.29 | 96.11 | 96.5 |
|  |  | 3.76 | 2.97 | 3.15 | 3.40 |
|  |  | 0.46 | 0.74 | 0.74 | 0.2 |

**Rpim*= Σhkl[1/(N-1)]^1/2^Σi|Ii(hkl) − ‹I(hkl)›|/ΣhklΣiIi(hkl), where N is the multiplicity measured.

***Rwork* = Σ ||*Fcalc*|−|*Fobs*|| / Σ|*Fobs*| × 100, where *Fcalc* and *Fobs* are the calculated and observed structure factor amplitudes, respectively. ****Rfree* is calculated for a randomly chosen 5% of the reflections for each dataset.


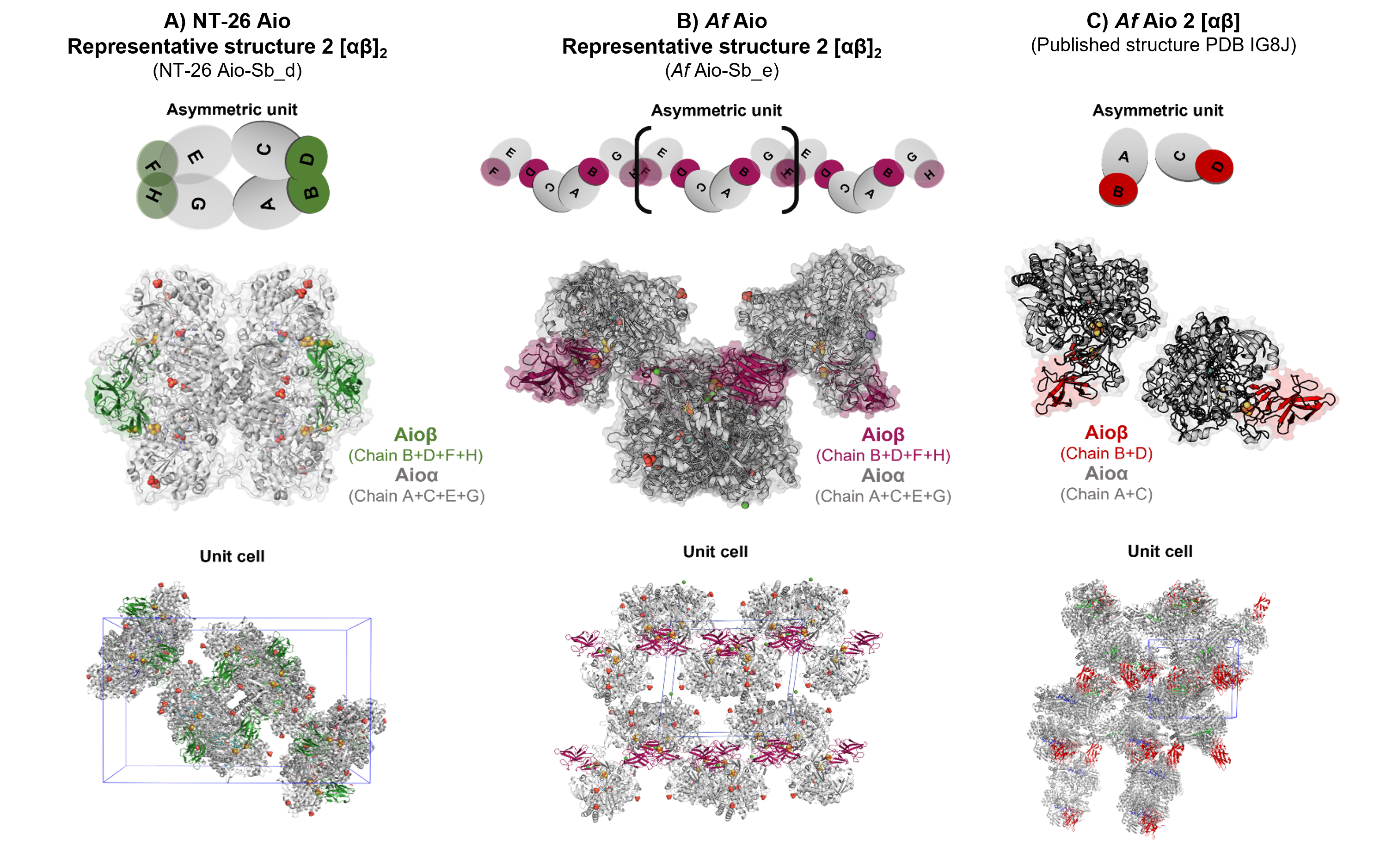


Figure S1. NT-26 and *Af* Aio heterotetrameric arrangement (NT-26 Aio-Sb_d, *Af* Aio-Sb_e and *Af* Aio PDB 1G8J [26]). Aio crystals contain 4 αβ heterodimers in the asymmetric unit – 2 αβ units interact directly and 2 other symmetry related molecules. **A)** NT-26 Aio complexes crystallized in P22_1_2_1_ SG - a=141.5, b=148.4, c=232.7 (Å) – as the previously deposited Aio-free structures PDB 4AAY/5NQD [25,27]. **B)** *Af* Aio crystal complexes belong to SG P1 - a=90.3, b=109.9, c=117.4 (Å); α=98.3, β=89.9, γ=96.6 (^o^) – as the P1 *Af* Aio structure 1G8K [26]. **C)** *Af* Aio structure PDB 1G8J [26]**,** with the SG P12_1_1 - a=96.71, b=114.25, c=108.98 (Å); α,γ=90^o^, β=112.4^o^.

Table S3. Analysis of protein-protein interactions of the reported Aio-free structures using PISA.

|  |  | **No. of**  **molecules AU** | **Buried Surface Area (Å^2^)** | | **Number of salt Bridges** | | **Number of H- bonds** | | **Interface Area (Å^2^)** | |
| --- | --- | --- | --- | --- | --- | --- | --- | --- | --- | --- |
| **Reported Aio ligand-free**  **structures** | **5NQD [28]** | 4 | 24362.5  (EF-AB) | 26358.9  (GH-CD) | 6  (E-A) | 6  (G-C) | 26  (E-A) | 22  (G-C) | 2368  (E-A) | 2336  (G-C) |
|  | **4AAY [26]** | 4 | 22346.6  (EF-CD) | 22140.1  (GH-AB) | 6  (E-C) | 9  (G-A) | 30  (E-C) | 26  (G-A) | 2539  (E-C) | 2465  (G-A) |
|  | **1G8K [27]** | 4 | 22377.9  (AB-CD) | 22626.0  (EF-GH) | 6  (G-E) | 6  (E-F) | 23  (G-E) | 24  (E-F) | 2288  (E-G) | 2243  (A-C) |
|  | **1G8J [27]** | 2 | 16661.5  (AB-CD) | - | 0  (A-C) | - | 9  (A-C) | - | 846  (A-C) | - |
| **This work: obtained**  **complexes structures** | ***Af* Aio–Sb_e** | 4 | 25007.0  (EF-GH) | 25710.3  (AB-CD) | 4  (G-E) | 4  (C-A) | 28  (G-E) | 26  (C-A) | 2350  (G-E) | 2319  (C-A) |
|  | **NT-26 Aio–Sb_d** | 4 | 26588.3  (EF-AB) | 26961.1  (GH-CD) | 7  (E-A) | 6  (G-C) | 25  (E-A) | 22  (G-C) | 2326  (E-A) | 2304  (G-C) |
|  | ***Af* Aio–As_d** | 4 | 23174.7  (AB-GH) | 23948.1  (CD-EF) | 2  (E-C) | 3  (A-G) | 23  (E-C) | 23  (A-G) | 2379  (E-C) | 2369  (A-G) |
|  | ***Af* Aio-As_c** | 4 | 23965.0  (AB-EF) | 23754.4  (CD-GH) | 4  (A-E) | 4  (C-G) | 27  (A-E) | 25  (C-G) | 2352  (E-A) | 2351  (C-G) |

Table S4. Specific activity of NT-26 Aio WT and mutant enzymes**.**

|  | **NT-26 Aio WT** | **NT-26 Aio D169A** | **NT-26 Aio E453A** |
| --- | --- | --- | --- |
| [DCPIPred] (μM^-1^.min^-1^) | 0.23 | 0.51 | 0.12 |
| Specific activity (μmol.min^-1^.mg^-1^) | 1.74 | 1.22 | 0.62 |
| Specific activity (%) | 100.00 ± 1.30 | 70.20 ± 5.60 | 35.60 ± 4.20 |

Table S5. Solvent-accessible surface area (Å^2^) of several conserved residues of NT-26 Aio-Sb_d structure. Areas were calculated in PyMOL [82].

|  | **Solvent-accessible surface area (A^2^)** | | |
| --- | --- | --- | --- |
| **Chain** | **Asp169** | **Glu453** | **His451** |
| **A** | 250.562 | 285.202 | 141.780 |
| **C** | 250.152 | 281.497 | 143.299 |
| **E** | 250.303 | 284.752 | 143.781 |
| **G** | 250.602 | 281.210 | 142.646 |

## Aio active site analysis

In *Af* Aio–As_c, atoms As, O1 and O2 were modelled with an occupancy of 0.2, 1.0 and 1.0, respectively, and have been refined with B factors similar to those of surrounding residues; In NT-26 Aio-Sb_d, Sb atom was modelled with an occupancy of *ca* 0.8 in all 4 α chains of the *Af* Aio enzyme, while the oxygen ligands were refined with full occupancy. The B factors of the 5 coordinating oxygen atoms (19.45 - 33.23 Å^2^) are also similar to those reported for the nearby atoms and water molecules in the NT-26 Aio-Sb_d structure (average B factor of 39.48 Å^2^). Similarly, As was also modelled with an occupancy of ca 0.4 and the B factors of the 5 coordinating oxygen atoms range from 23.85 - 41.90 Å^2^. In *Af* Aio–Sb_e all oxygen ligands of Sb (O1, O2, O3, O4 and Ox) were refined with an occupancy of 1.0 and Sb with occupancy 0.6 - 0.8. Analysis of the B factors for those oxygens (9.63 – 17.54 Å^2^; Table S5) show that they are in the same range as the surrounding atoms and water molecules.

Table S6. B factor analysis of all the atoms surrounding the Mo and As/Sb atoms and corresponding occupancy values in parenthesis**,** in all 4 molecules present in the asymmetric unit**.**

|  | |  | ***Af* Aio–As_c** | | | | **NT-26 Aio–Sb_d** | | | | ***Af* Aio–Sb_e** | | | | ***Af* Aio–As_d** | | | |
| --- | --- | --- | --- | --- | --- | --- | --- | --- | --- | --- | --- | --- | --- | --- | --- | --- | --- | --- |
|  | **polypeptide chain** | | **A** | **C** | **E** | **G** | **A** | **C** | **E** | **G** | **A** | **C** | **E** | **G** | **A** | **C** | **E** | **G** |
| **B (Å^2^) / (occ)** | | **O_x_** | 13.36  (1.00) | 11.53  (1.00) | 12.94  (1.00) | 12.32  (1.00) | 25.11  (1.00) | 19.45  (1.00) | 26.25  (1.00) | 27.84  (1.00) | 23.85  (1.00) | 28.22  (1.00) | 26.56  (1.00) | 25.46  (1.00) | 11.54  (1.00) | 11.91  (1.00) | 11.91  (1.00) | 10.31  (1.00) |
|  |  | **O_1_** | 15.68  (1.00) | 13.88  (1.00) | 14.77  (1.00) | 13.09  (1.00) | 29.98  (1.00) | 25.76  (1.00) | 28.50  (1.00) | 33.23  (1.00) | 36.32  (1.00) | 41.90  (1.00) | 37.37  (1.00) | 37.72  (1.00) | 14.11  (1.00) | 16.49  (1.00) | 14.93  (1.00) | 13.20  (1.00) |
|  |  | **O_2_** | 14.18  (1.00) | 12.89  (1.00) | 13.32  (1.00) | 12.36  (1.00) | 30.24  (0.80) | 27.08  (0.80) | 29.60  (0.80) | 28.57  (0.80) | 35.02  (1.00) | 41.89  (1.00) | 33.44  (1.00) | 38.04  (1.00) | 11.54  (1.00) | 13.27  (1.00) | 12.11  (1.00) | 16.33  (1.00) |
|  |  | **O_3_** | - | - | - | - | 22.19  (0.80) | 27.50  (0.80) | 27.61  (0.80) | 26.88  (0.80) | 28.91  (1.00) | 33.80  (1.00) | 28.38  (1.00) | 37.45  (1.00) | 11.42  (0.80) | 16.75  (1.00) | 16.66  (1.00) | 16.93  (1.00) |
|  |  | **O_4_** | - | - | - | - | 22.45  (0.80) | 29.82  (0.80) | 26.42  (0.80) | 27.58  (0.80) | 35.81  (1.00) | 38.58  (1.00) | 33.44  (1.00) | 34.86  (1.00) | 13.34  (1.00) | 18.93  (1.00) | 18.53  (1.00) | 19.33  (1.00) |
|  |  | **As/Sb** | 14.97  (0.20) | 13.23  (0.20) | 14.01  (0.20) | 12.66  (0.20) | 24.79  (0.80) | 27.33  (0.80) | 26.22  (0.80) | 25.66  (0.80) | 27.65  (0.38) | 29.94  (0.39) | 25.41  (0.37) | 29.85  (0.39) | 10.15  (0.80) | 11.01  (0.70) | 8.35  (0.60) | 8.60  (0.60) |
|  |  | **Mo** | 9.90  (1.00) | 10.30  (1.00) | 10.23  (1.00) | 9.86  (1.00) | 21.40  (1.00) | 24.56  (1.00) | 25.75  (1.00) | 22.86  (1.00) | 19.37  (1.00) | 18.39  (1.00) | 19.20  (1.00) | 19.66  (1.00) | 8.77  (0.95) | 12.39  (1.00) | 8.69  (0.90) | 11.22  (1.00) |
|  |  | **Average B – all chain (Å^2^)** | 13.69 | 14.30 | 14.18 | 13.95 | 25.05 | 28.76 | 28.36 | 27.11 | 13.74 | 15.58 | 14.72 | 15.24 | 14.03 | 15.97 | 14.99 | 15.50 |
|  |  | **Average B – waters (Å^2^)** | 21.93 | | | | 39.48 | | | | 29.6 | | | | 29.24 | | | |

Table S7. Bond lengths (in Å) around Mo and As/Sb atoms at the catalytic site of Aio structures in all 4 molecules present in the asymmetric unit**.**

|  |  | ***Af* Aio–As_c** | | | | **NT-26 Aio–Sb_d** | | | | ***Af* Aio–Sb_e** | | | | | ***Af* Aio–As_d** | | | |  |
| --- | --- | --- | --- | --- | --- | --- | --- | --- | --- | --- | --- | --- | --- | --- | --- | --- | --- | --- | --- |
|  | **polypeptide chain** | **A** | **C** | **E** | **G** | **A** | **C** | **E** | **G** | **A** | **C** | **E** | **G** | **A** | | **C** | **E** | **G** | |
| **Bond length (Å)** | **As/Sb - O_x_** | 1.73 | 1.76 | 1.77 | 1.73 | 2.08 | 1.84 | 1.90 | 2.05 | 2.10 | 2.12 | 2.16 | 2.04 | 1.71 | | 1.71 | 1.72 | 1.78 | |
|  | **As/Sb - O_1_** | 1.74 | 1.72 | 1.73 | 1.73 | 2.15 | 2.34 | 2.21 | 2.26 | 1.78 | 1.81 | 1.82 | 1.99 | 1.75 | | 1.74 | 1.83 | 1.74 | |
|  | **As/Sb - O_2_** | 1.86 | 1.88 | 1.85 | 1.84 | 2.00 | 1.97 | 1.95 | 1.99 | 2.05 | 1.92 | 2.03 | 1.91 | 1.74 | | 1.72 | 1.73 | 1.77 | |
|  | **As/Sb - O_3_** | - | - | - | - | 2.00 | 1.98 | 2.03 | 2.06 | 2.02 | 2.00 | 1.98 | 1.81 | 1.79 | | 1.79 | 1.79 | 1.77 | |
|  | **As/Sb - O_4_** | - | - | - | - | 1.95 | 2.00 | 2.01 | 1.91 | 1.88 | 1.99 | 1.88 | 1.99 | 1.71 | | 1.72 | 1.73 | 1.69 | |
|  | **Mo ^….^ As/Sb** | 3.28 | 3.32 | 3.36 | 3.31 | 3.25 | 3.34 | 3.34 | 3.26 | 3.54 | 3.55 | 3.52 | 3.51 | 3.08 | | 3.06 | 3.10 | 3.08 | |
|  | **Mo - O_x_** | 1.74 | 1.75 | 1.70 | 1.73 | 2.07 | 2.16 | 1.97 | 2.26 | 1.82 | 2.00 | 1.94 | 1.95 | 1.99 | | 1.97 | 1.97 | 1.97 | |
|  | **Mo - O_1_** | 3.79 | 3.68 | 3.78 | 3.79 | 2.29 | 1.87 | 1.94 | 2.10 | 3.22 | 2.29 | 2.67 | 3.05 | 1.99 | | 2.23 | 1.77 | 2.05 | |

Table S8. Bond lengths (in Å) between catalytic Mo and the coordinated sulfur atoms of the bis-MGD moieties of Aio-intermediate bound structures in all 4 molecules present in the asymmetric unit**.**

|  | | ***Af* Aio–As_c** | | | | **NT-26 Aio–Sb_d** | | | | ***Af* Aio–Sb_e** | | | | ***Af* Aio–As_d** | | | |  |
| --- | --- | --- | --- | --- | --- | --- | --- | --- | --- | --- | --- | --- | --- | --- | --- | --- | --- | --- |
| **polypeptide chain** | | **A** | **C** | **E** | **G** | **A** | **C** | **E** | **G** | **A** | **C** | **E** | **G** | **A** | **C** | **E** | **G** | |
| **Bond length (Å)** | **Mo-S_1_** | 2.32 | 2.34 | 2.33 | 2.33 | 2.36 | 2.31 | 2.34 | 2.38 | 2.37 | 2.37 | 2.35 | 2.37 | 2.31 | 2.27 | 2.26 | 2.30 | |
|  | **Mo-S_2_** | 2.37 | 2.36 | 2.37 | 2.36 | 2.41 | 2.35 | 2.41 | 2.29 | 2.44 | 2.42 | 2.49 | 2.40 | 2.45 | 2.40 | 2.39 | 2.39 | |
|  | **Mo-S_3_** | 2.35 | 2.28 | 2.30 | 2.28 | 2.37 | 2.38 | 2.41 | 2.43 | 2.33 | 2.35 | 2.37 | 2.37 | 2.31 | 2.34 | 2.35 | 2.34 | |
|  | **Mo-S_4_** | 2.40 | 2.41 | 2.39 | 2.41 | 2.46 | 2.48 | 2.46 | 2.46 | 2.40 | 2.41 | 2.40 | 2.45 | 2.39 | 2.40 | 2.47 | 2.37 | |


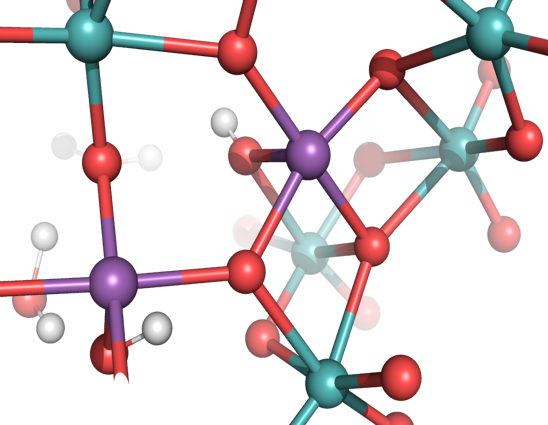


**Sb**

**Mo**

2.306

2.226

3.349

2.020

1.975

1.928

1.995

1.976

Figure S2. Sb-Mo polyoxometalate (CSD YORPUS [36]). In this complex, the Mo sits at 3.35 Å from Sb, and the distance between Sb and O atoms varies from 1.93 to 2.02 Å, as those found in the complexes described in this work. Distances in Å. Picture prepared using PyMOL [34].

**Figure S3.** A) *Af* Aio-As_c active site, chain E. Electron density map *2Fo-Fc*, shown in blue, contoured at 1σ; anomalous difference Fourier map, shown in magenta, contoured at 3σ. B) Plot of the theoretical atomic scattering factors (*f’* and *f’’* in [e]) of As, S and Mo as a function of X-ray energy (in eV) or wavelength (in Å); inset plot at the lower right corner with corresponding values of the theoretical number of electrons of anomalous scattering expected for fully occupied As and Mo atoms at the wavelength used for the different data collections reported (calculated from Ethan Merritt's website [online] . <http://skuld.bmsc.washington.edu/scatter/AS_form.html> (accessed June 22, 2023)).


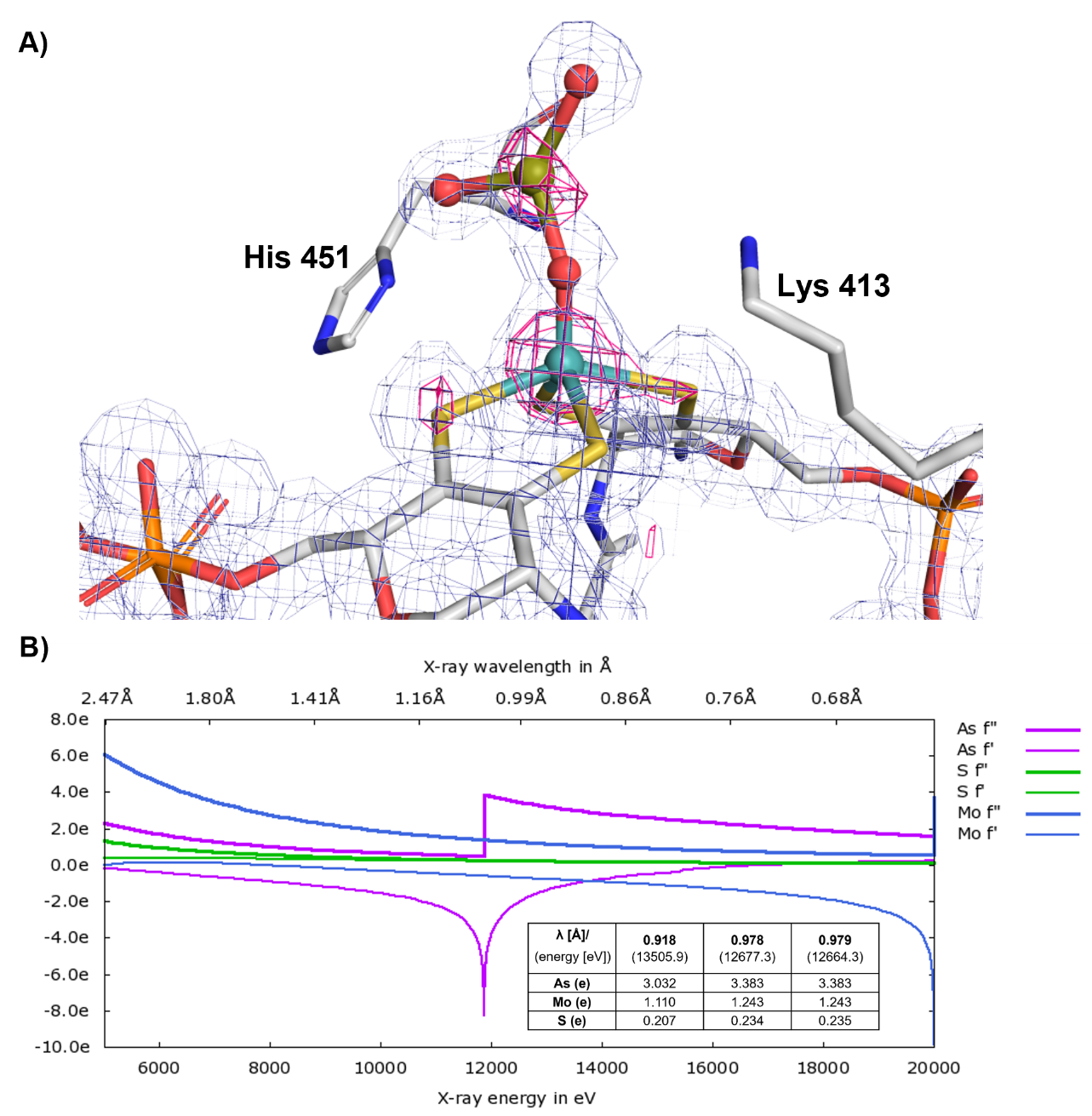


**DFT Supplementary Information**

##

Figure S4. Simplified molybdopterin cofactor model used in DFT calculations. The **A)** dithiolate model used in the calculations reproduces the pyran ring framework observed in **B)** the real molybdopterin cofactor.


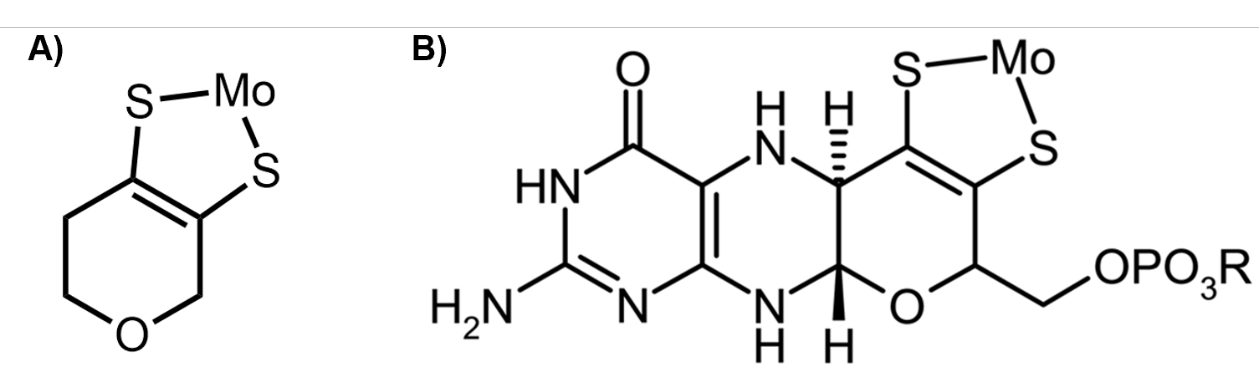


## Atomic coordinates of the optimized species

H_2_O

H 0.500966 -0.317001 -0.179542

O 0.101805 0.244067 0.494143

H -0.686871 0.580534 0.055098

As(OH)_3_

As 14.129713 -47.404896 -66.103731

O 13.777286 -48.998963 -66.844333

O 12.510482 -46.829874 -66.612076

O 15.029660 -46.730321 -67.515207

H 14.465531 -49.610998 -66.550014

H 12.283155 -46.085455 -66.039254

H 15.816572 -46.300092 -67.154284

AsO(OH)_3_

As 13.832692 -47.625382 -66.214771

O 13.632949 -49.117292 -67.060167

O 12.380245 -46.908975 -66.824646

O 14.016314 -47.657527 -64.608328

O 15.137927 -46.885440 -67.088876

H 14.363998 -49.713698 -66.836712

H 12.243596 -46.053423 -66.388945

H 15.616679 -46.300863 -66.481454

A

Mo -0.627179 0.569367 -1.662815

O 0.304423 1.376796 -2.849662

H 3.170325 -1.711065 1.137781

C 2.691669 -2.450172 0.489271

O 2.330873 -3.540767 1.304877

C 1.507296 -1.822380 -0.211625

S 1.712111 -0.337922 -0.972163

C 0.288002 -2.511773 -0.274344

S -1.082266 -1.818200 -0.968457

C 0.179738 -3.899915 0.295414

C 1.554921 -4.480431 0.576712

C -2.036977 1.733466 2.970820

O -1.210571 2.315747 3.950914

C -1.363068 1.606225 1.623225

S -2.099993 0.657711 0.454517

C -0.204245 2.351133 1.345328

S 0.544442 2.267436 -0.157232

C 0.373272 3.250090 2.403972

C -0.656069 3.536302 3.484263

O -2.170951 0.392991 -2.381637

H -2.956385 2.327015 2.835477

H -2.327664 0.745628 3.338280

H 0.734507 4.182123 1.958752

H 1.239852 2.746569 2.851557

H -0.188203 4.024295 4.341768

H -1.452733 4.189718 3.099610

H -0.377361 -4.547039 -0.389854

H -0.396341 -3.847912 1.228092

H 1.473308 -5.377017 1.194912

H 2.066888 -4.745566 -0.359799

H 3.427379 -2.760454 -0.270555

B

Mo 14.459401 -48.815648 -62.536104

As 12.499169 -46.012938 -65.376076

O 15.920722 -48.834313 -63.393812

H 15.540773 -51.130648 -57.780494

C 15.909275 -51.697102 -58.642175

O 15.484522 -53.030518 -58.471924

C 15.369124 -51.094149 -59.919442

S 15.465949 -49.373723 -60.096132

C 14.796661 -51.850611 -60.888543

S 13.944356 -51.190040 -62.257434

C 14.773114 -53.346399 -60.756068

C 15.712924 -53.793034 -59.647406

C 12.821858 -46.761538 -58.210774

O 13.264698 -45.504228 -57.752143

C 13.382364 -47.086136 -59.577414

S 13.279457 -48.728069 -60.118427

C 13.982202 -46.149018 -60.352540

S 14.864116 -46.513956 -61.810932

C 14.011099 -44.714872 -59.908087

C 13.063348 -44.502770 -58.737676

O 14.274164 -46.095725 -65.660258

O 11.978181 -47.394867 -66.385119

O 13.134494 -48.678711 -63.581958

O 12.104008 -44.784229 -66.627321

H 14.447375 -46.100076 -66.613867

H 12.366969 -47.319827 -67.270254

H 12.049724 -43.924981 -66.185929

H 11.721259 -46.796930 -58.240347

H 13.163953 -47.505518 -57.483646

H 13.732179 -44.061858 -60.742779

H 15.037771 -44.451337 -59.624884

H 13.257912 -43.541163 -58.257854

H 12.019130 -44.517884 -59.081413

H 15.064528 -53.807845 -61.706247

H 13.746394 -53.670138 -60.544816

H 15.530763 -54.838367 -59.389003

H 16.759937 -53.688591 -59.966691

H 17.009435 -51.639943 -58.640107

TS_BC_

Mo 15.200637 -48.810300 -62.598379

As 13.239866 -46.691616 -64.261109

O 16.743712 -48.754904 -63.266657

H 15.136266 -51.125170 -57.907140

C 15.627898 -51.730527 -58.676198

O 15.076563 -53.025927 -58.591660

C 15.403928 -51.116461 -60.039413

S 15.625839 -49.396693 -60.172765

C 15.004447 -51.842994 -61.116693

S 14.540623 -51.160684 -62.656878

C 14.857310 -53.331420 -60.976552

C 15.494271 -53.825674 -59.687798

C 12.795082 -46.647709 -58.698752

O 13.003378 -45.293319 -58.370150

C 13.481785 -47.025563 -59.990690

S 13.499355 -48.716142 -60.416282

C 13.989328 -46.084768 -60.819512

S 15.036235 -46.471346 -62.176137

C 13.755627 -44.627143 -60.563498

C 12.711061 -44.448083 -59.473138

O 14.215212 -46.583103 -65.742792

O 11.691742 -47.392282 -64.764180

O 13.887588 -48.510907 -63.874336

O 12.543191 -45.016531 -64.262285

H 14.008616 -45.737484 -66.169751

H 11.100104 -46.670933 -65.025267

H 13.230817 -44.392083 -63.987272

H 11.718413 -46.870305 -58.771270

H 13.207927 -47.244420 -57.878510

H 13.415874 -44.152528 -61.493097

H 14.701983 -44.147661 -60.284909

H 12.718087 -43.422349 -59.098767

H 11.707761 -44.671928 -59.862921

H 15.312044 -53.831555 -61.838985

H 13.788121 -53.578093 -60.995226

H 15.180233 -54.849335 -59.472024

H 16.590850 -53.806975 -59.769411

H 16.705535 -51.756786 -58.447691

C

Mo 14.660275 -48.881137 -61.722488

As 12.991633 -47.342109 -64.345306

O 16.221990 -49.045781 -62.332547

H 16.009474 -51.952737 -57.939237

C 16.231859 -52.261716 -58.966694

O 15.936266 -53.640406 -59.054835

C 15.432943 -51.433137 -59.939714

S 15.211742 -49.742653 -59.561924

C 14.792256 -52.009488 -60.989810

S 13.726196 -51.085001 -62.031677

C 14.899030 -53.489098 -61.236589

C 16.019334 -54.087287 -60.399860

C 12.037678 -45.735532 -58.934202

O 12.537559 -44.491025 -58.494928

C 12.962753 -46.431143 -59.905003

S 12.691863 -48.076539 -60.235915

C 13.994471 -45.729850 -60.498172

S 15.160592 -46.539608 -61.441155

C 14.152733 -44.248760 -60.268955

C 12.911521 -43.681983 -59.600095

O 14.443329 -46.786770 -65.078336

O 11.883634 -47.942002 -65.505168

O 13.327789 -48.649477 -63.397456

O 12.214379 -46.033150 -63.541385

H 14.283886 -45.961964 -65.568949

H 11.652496 -47.247643 -66.144153

H 12.781771 -45.243993 -63.558954

H 11.050912 -45.604494 -59.408244

H 11.899653 -46.357125 -58.044363

H 14.328873 -43.735640 -61.220802

H 15.032818 -44.067012 -59.639653

H 13.104572 -42.679568 -59.210630

H 12.079160 -43.623023 -60.317361

H 15.091761 -53.688684 -62.296685

H 13.940437 -53.967004 -60.993466

H 15.947312 -55.177507 -60.376272

H 16.997891 -53.813019 -60.822315

H 17.308469 -52.092635 -59.136800

C’

As 13.595618 -46.660939 -66.376146

Mo 13.335712 -48.426899 -63.590020

O 14.411369 -47.433254 -65.153377

H 17.522753 -51.508305 -63.416852

C 16.592096 -52.010347 -63.131614

O 16.870589 -52.736058 -61.950470

C 15.489354 -51.005979 -62.924320

S 15.282653 -49.749211 -64.119943

C 14.678950 -51.057211 -61.857748

S 13.426917 -49.844074 -61.648566

C 14.816446 -52.128154 -60.813266

C 15.684563 -53.251571 -61.362868

C 11.328558 -46.492979 -59.675960

O 12.048982 -45.841218 -58.650882

C 12.171824 -46.724951 -60.907307

S 11.513473 -47.686416 -62.171353

C 13.376072 -46.100605 -61.074603

S 14.338711 -46.425766 -62.465392

C 13.855834 -45.094313 -60.065209

C 12.714861 -44.688495 -59.146797

O 12.643004 -47.661660 -67.388500

O 12.487907 -45.525550 -65.728361

O 12.062853 -49.052447 -64.521842

O 14.692615 -45.900743 -67.451884

H 13.179013 -48.399159 -67.726596

H 12.225917 -44.887058 -66.413676

H 15.320441 -45.354800 -66.948369

H 10.433168 -45.907381 -59.943186

H 10.989344 -47.449329 -59.265633

H 14.258878 -44.213301 -60.577999

H 14.678292 -45.523846 -59.479739

H 13.088306 -44.141283 -58.277836

H 12.000413 -44.045288 -59.682299

H 13.834905 -52.530506 -60.536480

H 15.259877 -51.711730 -59.899351

H 16.000697 -53.927737 -60.564495

H 15.121739 -53.832562 -62.108962

H 16.321196 -52.698176 -63.950400

TS_C’D_

As 13.427142 -46.622781 -66.198927

Mo 13.478207 -48.717069 -63.022384

O 14.520083 -47.273462 -65.186007

H 17.784449 -51.311461 -62.384354

C 16.870563 -51.864780 -62.626336

O 16.861426 -53.019343 -61.813057

C 15.661053 -50.995906 -62.398490

S 15.777408 -49.316331 -62.850618

C 14.553378 -51.481093 -61.781306

S 13.196505 -50.427427 -61.437358

C 14.499009 -52.901796 -61.294354

C 15.625495 -53.709535 -61.922385

C 11.353769 -45.992787 -59.760741

O 12.056252 -45.181142 -58.847391

C 12.251314 -46.547417 -60.844749

S 11.635843 -47.804972 -61.820299

C 13.493919 -45.992674 -61.092395

S 14.444809 -46.524962 -62.410203

C 13.976638 -44.837828 -60.261139

C 12.817097 -44.190236 -59.521624

O 12.625028 -47.681251 -67.306849

O 12.085298 -45.948306 -65.343631

O 12.921128 -49.546087 -64.382159

O 14.050081 -45.400704 -67.256577

H 13.284507 -48.203498 -67.791378

H 11.458540 -45.551496 -65.970832

H 14.743258 -44.909189 -66.786569

H 10.533647 -45.426699 -60.232822

H 10.909727 -46.807860 -59.182729

H 14.485688 -44.102062 -60.892691

H 14.715743 -45.208477 -59.539405

H 13.183074 -43.498511 -58.759602

H 12.175083 -43.634371 -60.220569

H 13.536613 -53.360169 -61.547428

H 14.582475 -52.917088 -60.199585

H 15.751565 -54.665415 -61.408440

H 15.403966 -53.909755 -62.981474

H 16.924119 -52.145357 -63.691441

D

As 13.638518 -46.727455 -66.616829

Mo 13.577415 -48.752011 -62.641962

O 15.056175 -47.113208 -65.939358

H 17.804847 -51.391631 -62.057052

C 16.898743 -51.884414 -62.424998

O 16.826271 -53.143525 -61.792902

C 15.693961 -51.025304 -62.138852

S 15.862212 -49.308593 -62.350143

C 14.537376 -51.579463 -61.659818

S 13.173121 -50.578927 -61.235018

C 14.442934 -53.056466 -61.403254

C 15.593300 -53.786003 -62.079188

C 11.273524 -45.817723 -59.660863

O 11.913097 -44.903107 -58.799951

C 12.239562 -46.457403 -60.632506

S 11.703703 -47.831139 -61.504219

C 13.472892 -45.897739 -60.871325

S 14.526075 -46.556359 -62.056525

C 13.897266 -44.652935 -60.146892

C 12.688949 -43.965265 -59.529938

O 12.643250 -48.006858 -67.221659

O 12.578063 -45.991895 -65.464041

O 13.170880 -49.396706 -64.138813

O 13.719050 -45.678204 -67.996196

H 13.122750 -48.482542 -67.918561

H 11.733718 -45.765855 -65.885676

H 14.459429 -45.061576 -67.879724

H 10.472327 -45.320146 -60.231897

H 10.811619 -46.579449 -59.026652

H 14.413866 -43.971518 -60.831214

H 14.613072 -44.925220 -59.360976

H 13.004527 -43.195232 -58.822579

H 12.074046 -43.494238 -60.310531

H 13.488684 -53.448025 -61.771732

H 14.463358 -53.231942 -60.319504

H 15.674922 -54.809424 -61.706276

H 15.434765 -53.822128 -63.167127

H 17.003631 -52.004668 -63.516047

E

Mo 2.191417 5.478607 0.546129

C -0.777996 1.763535 1.159618

O -0.477155 0.408571 0.895555

C 0.404046 2.660281 0.889277

S 0.086123 4.369973 0.699283

C 1.646762 2.168076 0.767703

S 2.982364 3.235778 0.378096

C 1.929323 0.698115 0.896889

C 0.727992 0.012295 1.533134

O 2.524064 5.921545 2.133865

C 4.772414 8.15595 0.183477

O 4.479413 9.507413 0.427653

C 3.596172 7.373138 -0.354418

S 3.944328 5.808324 -0.98992

C 2.29166 7.877929 -0.17899

S 0.878425 7.004007 -0.664934

C 2.102076 9.184167 0.541817

C 3.351888 9.626779 1.280646

H 5.13213 7.653989 1.09629

H 5.577109 8.123702 -0.557745

H 1.254185 9.109443 1.228584

H 1.834634 9.926212 -0.222486

H 3.274072 10.676906 1.568078

H 3.501077 9.023967 2.187736

H -1.107506 1.887333 2.204571

H -1.619734 2.031377 0.512454

H 2.816563 0.524495 1.516366

H 2.137444 0.268151 -0.091289

H 0.798036 -1.072739 1.423996

H 0.678674 0.252881 2.605563

F

Mo -0.230500 -0.097869 -0.261677

H 0.272577 2.428210 -2.779456

O 0.727393 1.662924 -3.149551

H 3.932166 -2.872128 -0.020953

C 3.003146 -3.289531 -0.423095

O 2.905539 -4.615593 0.046795

C 1.831925 -2.440098 -0.002163

S 2.050497 -0.717851 -0.003204

C 0.664370 -3.014576 0.424623

S -0.672308 -2.032407 0.971896

C 0.533336 -4.508999 0.503008

C 1.646761 -5.180962 -0.286363

C -2.645460 2.590642 2.863736

O -2.043739 3.408781 3.841352

C -1.633315 2.024967 1.893180

S -2.137999 0.759036 0.856698

C -0.375741 2.568763 1.784710

S 0.735096 1.991172 0.608095

C 0.027388 3.728717 2.647875

C -1.204564 4.388318 3.249162

H 0.181265 0.918915 -2.861002

O -0.659009 -0.612392 -1.807222

H -3.407053 3.152931 2.298771

H -3.152060 1.781937 3.397822

H 0.601448 4.456470 2.064568

H 0.684329 3.365757 3.448425

H -0.920078 5.083092 4.042394

H -1.759202 4.942871 2.478394

H -0.439611 -4.829548 0.115734

H 0.575381 -4.815231 1.556658

H 1.703645 -6.244791 -0.045602

H 1.464819 -5.076030 -1.366191

H 3.079062 -3.272097 -1.523115

TS_FG_

Mo -0.253344 -0.009102 -0.556229

H -0.053639 2.514890 -2.390813

O 0.539727 1.761519 -2.501468

H 3.928435 -2.808507 -0.081362

C 3.007647 -3.284932 -0.434595

O 2.918820 -4.538518 0.208692

C 1.822822 -2.402802 -0.137009

S 2.025068 -0.684715 -0.339862

C 0.669933 -2.920564 0.367196

S -0.662655 -1.872048 0.802855

C 0.544108 -4.390834 0.649733

C 1.663958 -5.152802 -0.043085

C -2.623039 2.531142 2.716469

O -2.008984 3.219219 3.782630

C -1.614363 2.008596 1.717237

S -2.148654 0.865306 0.570286

C -0.327517 2.510642 1.677598

S 0.765191 2.026231 0.455174

C 0.098782 3.559693 2.664650

C -1.115827 4.213303 3.304499

H 0.093904 1.203666 -3.153021

O -0.775109 -0.648223 -2.029414

H -3.337371 3.186924 2.191709

H -3.186413 1.703859 3.156465

H 0.723330 4.312725 2.172691

H 0.715552 3.085208 3.438576

H -0.820238 4.815409 4.166570

H -1.629373 4.863908 2.581647

H -0.424316 -4.768035 0.303418

H 0.582877 -4.556946 1.734445

H 1.730837 -6.174966 0.336709

H 1.480523 -5.194873 -1.127125

H 3.098831 -3.417471 -1.525466

G

Mo -0.252873 0.021674 -0.592781

H 0.099673 2.380634 -2.337897

O 0.626493 1.570086 -2.331037

H 3.924241 -2.816914 -0.275839

C 2.980293 -3.295907 -0.557422

O 2.923129 -4.528081 0.129985

C 1.820945 -2.395766 -0.216430

S 2.028893 -0.684565 -0.486888

C 0.698534 -2.884455 0.369701

S -0.604941 -1.807397 0.837231

C 0.582089 -4.341880 0.717419

C 1.649912 -5.137848 -0.018282

C -2.652526 2.535437 2.697216

O -2.055336 3.207039 3.783327

C -1.626460 2.025961 1.708471

S -2.140476 0.908117 0.531964

C -0.335755 2.522492 1.700248

S 0.784915 2.030082 0.509772

C 0.070655 3.562649 2.705548

C -1.154826 4.207530 3.333071

H 0.339303 1.091179 -3.121978

O -0.871354 -0.639995 -2.020909

H -3.358084 3.199942 2.171856

H -3.223184 1.701845 3.115530

H 0.701568 4.321650 2.231150

H 0.676520 3.081699 3.484026

H -0.874254 4.798955 4.207492

H -1.656886 4.867150 2.610273

H -0.408145 -4.725891 0.449032

H 0.691491 -4.468357 1.802779

H 1.734319 -6.146983 0.391940

H 1.396594 -5.215940 -1.086178

H 3.005037 -3.463740 -1.647088

TS_C’H_

As 13.425659 -47.345591 -66.156081

Mo 13.426212 -48.288108 -63.127061

O 14.456825 -47.404161 -64.841154

H 17.717339 -51.016196 -62.205014

C 16.849694 -51.533599 -62.628242

O 16.752055 -52.776215 -61.961939

C 15.607546 -50.698271 -62.460113

S 15.743831 -48.978367 -62.699650

C 14.440161 -51.248345 -62.056015

S 13.045847 -50.224501 -61.785409

C 14.340725 -52.711050 -61.723400

C 15.545259 -53.445150 -62.295546

C 11.448716 -46.086979 -59.441929

O 12.164464 -45.315124 -58.503109

C 12.295850 -46.459007 -60.637124

S 11.639964 -47.558902 -61.781577

C 13.485648 -45.823750 -60.890976

S 14.369074 -46.182168 -62.320645

C 13.964249 -44.719221 -59.991846

C 12.820054 -44.219492 -59.124587

O 13.089584 -48.806003 -67.011983

O 11.917487 -46.543061 -65.904636

O 12.369726 -48.746627 -64.418739

O 14.236827 -46.430736 -67.382704

H 13.759087 -48.910556 -67.708989

H 11.814298 -45.873773 -66.602523

H 14.857301 -45.817222 -66.955925

H 10.552358 -45.545073 -59.786676

H 11.115222 -46.987588 -58.918214

H 14.374309 -43.897783 -60.590051

H 14.779342 -45.093853 -59.360313

H 13.191817 -43.577341 -58.322892

H 12.102129 -43.645706 -59.728860

H 13.423435 -53.146046 -62.135054

H 14.297525 -52.842221 -60.633931

H 15.620055 -54.452996 -61.879697

H 15.454459 -53.526300 -63.389273

H 17.047268 -51.687719 -63.702423

H

As 13.217605 -47.801361 -65.878007

Mo 13.580742 -48.163596 -63.016574

O 14.509170 -47.569669 -64.707317

H 17.697813 -50.938502 -61.974063

C 16.876363 -51.383599 -62.544167

O 16.717254 -52.706655 -62.083161

C 15.624205 -50.560575 -62.381503

S 15.773377 -48.853596 -62.344105

C 14.397152 -51.171128 -62.209742

S 12.985008 -50.216115 -61.964720

C 14.301363 -52.668543 -62.127432

C 15.571578 -53.311656 -62.663405

C 11.416793 -45.946662 -59.406901

O 12.094074 -45.176566 -58.438872

C 12.324076 -46.358842 -60.542244

S 11.740848 -47.481856 -61.697491

C 13.538363 -45.744095 -60.731678

S 14.491162 -46.156581 -62.095637

C 13.989944 -44.635202 -59.825568

C 12.806047 -44.101892 -59.034139

O 12.942069 -49.326100 -66.691348

O 11.926740 -46.630913 -66.068171

O 12.329975 -48.497467 -64.413424

O 14.173989 -47.171943 -67.259250

H 13.416444 -49.292199 -67.536294

H 12.166718 -46.075325 -66.825997

H 14.886096 -46.622745 -66.903185

H 10.556591 -45.391251 -59.815624

H 11.031206 -46.830679 -58.890908

H 14.450232 -43.830219 -60.408978

H 14.757340 -45.017709 -59.141170

H 13.143999 -43.456590 -58.220279

H 12.136609 -43.522854 -59.686823

H 13.434027 -53.031089 -62.688086

H 14.150057 -52.954015 -61.078211

H 15.606336 -54.371636 -62.402664

H 15.614627 -53.219309 -63.758660

H 17.165405 -51.376067 -63.608500

## References

(25) Warelow, T. P.; Oke, M.; Schoepp-Cothenet, B.; Dahl, J. U.; Bruselat, N.; Sivalingam, G. N.; Leimkühler, S.; Thalassinos, K.; Kappler, U.; Naismith, J. H.; et al. The Respiratory Arsenite Oxidase: Structure and the Role of Residues Surrounding the Rieske Cluster. PLoS One 2013, 8 (8), e72535.

(26) Ellis, P. J.; Conrads, T.; Hille, R.; Kuhn, P. Crystal Structure of the 100 kDa Arsenite Oxidase from *Alcaligenes faecalis* in Two Crystal Forms at 1.64 Å and 2.03 Å. *Structure*. 2001, 9 (2), 125-132.

(27) Watson, C.; Niks, D.; Hille, R.; Vieira, M.; Schoepp-Cothenet, B.; Marques, A. T.; Romão, M. J.; Santos-Silva, T.; Santini, J. M. Electron Transfer through Arsenite Oxidase: Insights into Rieske Interaction with Cytochrome c. Biochim. *Biophys. Acta - Bioenerg*. 2017, 1858 (10), 865–872.

(36) Bouallegui, T.; Harchani, A.; Dege, N.; Haddad, A.; Ayed, B. Synthesis, characterization, hirshfeld surface and theoretical properties of a new non-centrosymmetric inorganic/organic material: (C_7_H_12_N_2_)_7_ (β-SbMo_6_O_24_ )_2_·8H_2_O. J. Mol. Struct. 2018, 1166, 195-201.

(34) DeLano, W. L. Pymol: An open-source molecular graphics tool. *CCP4 Newsletter On Protein Crystallography*. 2002, *40*, 82-9.
